# Machine Learning Approaches to Classify Self-Reported Rheumatoid Arthritis Health Scores Using Activity Tracker Data: Longitudinal Observational Study

**DOI:** 10.2196/43107

**Published:** 2023-06-26

**Authors:** Kaushal Rao, William Speier, Yiwen Meng, Jinhan Wang, Nidhi Ramesh, Fenglong Xie, Yujie Su, W Benjamin Nowell, Jeffrey R Curtis, Corey Arnold

**Affiliations:** 1 Department of Radiological Sciences University of California, Los Angeles Los Angeles, CA United States; 2 Division of Clinical Immunology and Rheumatology University of Alabama at Birmingham Birmingham, AL United States; 3 Global Healthy Living Foundation Upper Nyack, NY United States

**Keywords:** rheumatoid arthritis, rheumatic, rheumatism, Fitbit, classification, physical data, digital health, activity tracker, mobile health, machine learning, model, patient reported, outcome measure, PROMIS, nonclinical monitoring, mHealth, tracker, wearable, arthritis, mobile phone

## Abstract

**Background:**

The increasing use of activity trackers in mobile health studies to passively collect physical data has shown promise in lessening participation burden to provide actively contributed patient-reported outcome (PRO) information.

**Objective:**

The aim of this study was to develop machine learning models to classify and predict PRO scores using Fitbit data from a cohort of patients with rheumatoid arthritis.

**Methods:**

Two different models were built to classify PRO scores: a random forest classifier model that treated each week of observations independently when making weekly predictions of PRO scores, and a hidden Markov model that additionally took correlations between successive weeks into account. Analyses compared model evaluation metrics for (1) a binary task of distinguishing a normal PRO score from a severe PRO score and (2) a multiclass task of classifying a PRO score state for a given week.

**Results:**

For both the binary and multiclass tasks, the hidden Markov model significantly (*P*<.05) outperformed the random forest model for all PRO scores, and the highest area under the curve, Pearson correlation coefficient, and Cohen κ coefficient were 0.750, 0.479, and 0.471, respectively.

**Conclusions:**

While further validation of our results and evaluation in a real-world setting remains, this study demonstrates the ability of physical activity tracker data to classify health status over time in patients with rheumatoid arthritis and enables the possibility of scheduling preventive clinical interventions as needed. If patient outcomes can be monitored in real time, there is potential to improve clinical care for patients with other chronic conditions.

## Introduction

Rheumatoid arthritis (RA) is a progressive autoimmune disease that causes irreversible joint damage, decline in functional status, and premature mortality, and is one of the most serious rheumatologic conditions in high-income countries [1]. Over the past decade, there has been a trend toward mobile health (mHealth) interventions that collect greater amounts of passive and active data, as well as improve treatment results, for people living with chronic illnesses such as RA [2]. For instance, web-based patient self-assessments were found to strongly correlate with rheumatologist assessments of RA activity, which justified further exploration of their use as cost-effective tools to monitor RA activity between outpatient visits [3]. In addition, due to their rapidly increasing ownership, smartphones and low-cost consumer devices have become widely used as noninvasive self-assessment and rehabilitation tools for heart failure, diabetes, and pulmonary disease [4-6]. In fact, in one study, a majority of patients expressed willingness to even pay for a RA self-management app [7]. Although the use of devices and apps for outpatient care has great potential and telehealth solutions have greatly motivated interest in remote health care delivery, device fatigue and poor usability pose threats to consistent and accurate recording of data that requires active engagement with an app that requires patients to answer questions [5,8]. Another trend that has emerged is the use of activity trackers and artificial intelligence to further streamline the process of passive data collection and machine learning for clinical applications; research has shown that the use of continuous-time activity trackers can be effective in a telemonitoring application with a high level of adherence and low attrition [9]. Activity trackers in general have also been shown to promote self-care, habit formation, and goal reinforcement, all of which foster physical activity and long-term well-being [10,11]. Furthermore, activity tracker–derived data collected during one study correlated significantly with clinically relevant patient-reported outcomes (PROs), further justifying the use of activity trackers to identify patients in need of clinical intervention [9,12].

In this study, patient health scores were quantified using Patient-Reported Outcomes Measurement Information System (PROMIS) surveys. PROMIS is a National Institutes of Health initiative devoted to developing and validating better measurement tools for assessing patients’ pain, fatigue, sleep disturbance, physical function, and other domains of health [13]. The usage of PROMIS surveys increased over the past decade, largely due to their low barriers to completion and effectiveness in measuring important patient-centered outcomes in clinical care in an unbiased manner [14-16]. Numerous studies have now established actionable PROMIS score thresholds (distinguishing normal or mild from moderate or severe symptoms). The use of PROMIS measures in primary care has been shown to improve patient-provider communication by giving patients a voice and optimizing clinical decision-making [17]. Given that there are barriers that may limit the feasibility of continually requiring patients to fill out PROs over time, passive data collected from health activity trackers may provide an important complement to PRO scores and other clinical data. As an additional helpful tool in this setting, machine learning algorithms have been widely used in clinical research for tasks such as disease detection and outcome prediction, and statistical models such as random forest (RF) and gradient-boosted regression trees have previously proven useful in improving the risk prediction accuracy for conditions such as cardiovascular diseases [18,19]. These kinds of traditional machine learning models are useful in making isolated decisions but do not account for cases where trends over time can influence the outcome. Hidden Markov models (HMMs), on the other hand, are well-established temporal models that are effective in using sequential data to predict events such as patient state changes and disease progression over time [20].

In this analysis, we explored the usage of several machine learning models to classify PRO scores over time in a study of patients with RA [21]. More specifically, our goal was to quantify the agreement between PRO scores and passively collected data from Fitbit Versa (Fitbit Inc), a commercially available activity tracker. If we found that tracking physical metrics over time using activity trackers was meaningfully correlated with key components of patients’ health, reliance on actively collected self-reported health scoring could be diminished, reducing participant burden to complete PRO measures as frequently. In a prior study, wearable activity trackers were used to passively measure changes that could be used to provide estimates of the incidence and duration of gout flares [22]. A related study trained baseline machine learning models and HMMs with activity tracker–derived data in order to predict PRO scores in a cohort of patients with stable ischemic heart disease and found that the HMM outperformed the other baseline models for a majority of PRO scores [23]. We hoped to obtain similar results from our study, and herein we describe our approach to data preprocessing, model construction, and resulting analyses.

## Methods

### Data Acquisition

The Digital Tracking of Arthritis Longitudinally (DIGITAL) study was an ancillary study of the ArthritisPower registry (Advarra Institutional Review Board [IRB] protocol number 00026788). A cohort of 470 eligible patients with RA was recruited by researchers at the Global Healthy Living Foundation and the University of Alabama at Birmingham, and 278 (59.1%) of them qualified for participation in the main study after successfully meeting adherence thresholds during an initial 2-week lead-in period. The main study followed the 278 qualifying patients for 12 weeks (84 days), and each patient was given a Fitbit Versa 2 to record physical metrics such as heart rate, physical activity, calories burned, and sleep progression. All collected data, including various PRO surveys that patients were asked to fill out on a weekly basis, were inputted by patients into a study-specific app (the ArthritisPower registry, with a custom workflow unique to this study). Data were analyzed and stored in the Health Insurance Portability and Accountability Act–compliant Amazon Web Services cloud and analyzed at the University of Alabama at Birmingham and the University of California, Los Angeles (UCLA). Data were deidentified prior to being made available at UCLA. Analyses were done to summarize trends between and within participants over time, and descriptive statistics quantified adherence or compliance rates for the various measures and technology as well as any differences in the enrolled populations and those compliant with the study protocol. The mean age of the patient cohort was 50.2 (SD 11.1) years, the mean number of years since RA diagnosis was 9.4 (SD 10.1), and 91.7% (n=255) of subjects identified as female. Patient features included demographics, race or ethnicity, gender, age, years since RA diagnosis, comorbidities, and medications, and some of these data were also used for training our machine learning models. Based on adherence criteria we describe later in the Data Imputation and Preprocessing section, we excluded 59 of these 278 patients, resulting in 219 patients in the final analytic data set.

### Activity Tracker Data

The Fitbit Versa 2 is a commercially available activity tracker whose algorithms have been shown to accurately track metrics relating to physical activity, sleep, and energy expenditure [24-27]. Whenever the Fitbit Versa 2 was worn during the clinical phase of the study, it passively collected patient metrics and was synced to the ArthritisPower app continuously, but at a minimum, occurred every 5 days. Overall, there were 15 distinct physical features that were used to train our machine learning models, and the respective feature means and SDs among patients using all available data are depicted in Table 1. To account for noise and redundancy, each activity tracker–derived feature was aggregated on a daily level (ie, a 24-hour interval, from 8 PM on one day to 8 PM on the next day) based on the data collected with second- or minute-level precision.

**Table 1:** Daily-aggregated features from Fitbit across patients.

Feature			Value, mean (SD)
**Sleep metrics**			
	Daily total sleep (minutes)	445.01 (130.02)	
	Daily total light sleep (minutes)	261.81 (78.14)	
	Daily total REM^a^ sleep (minutes)	84.89 (37.55)	
	Daily total deep sleep (minutes)	61.79 (27.57)	
	Daily total wake (minutes awake during a sleep episode)	24.21 (15.28)	
**Physical metrics**			
	Daily total steps	5775.66 (4177.02)	
	Daily total calories	2211.15 (615.05)	
	Daily total wearing (minutes)	1259.68 (411.19)	
	Daily minimum heart rate (beats per minute)	66.25 (10.32)	
	Daily maximum heart rate (beats per minute)	97.51 (12.51)	
**Activity metrics**			
	Daily total activity (minutes)	246.78 (117.55)	
	Daily light activity (minutes)	226.93 (102.60)	
	Daily moderate activity (minutes)	22.53 (24.57)	
	Daily heavy activity (minutes)	24.71 (54.41)	
	Daily sedentary activity (minutes)	1201.98 (127.03)	

^a^REM**:** rapid eye movement.

### PRO Measures

PROMIS questionnaires are a library of instruments developed and validated to measure domains of physical and mental health [16]. In this study, patients were asked to actively input information regarding 6 PROMIS scores on a weekly basis through several questionnaires on the study-specific ArthritisPower app [21]. Within the ArthritisPower app, PROMIS computer adaptive testing instruments were used to record scores relating to pain, physical function, fatigue, sleep disturbance, and satisfaction with participation in discretionary social activities (“social activity” for concision). The *t* score metric was used to standardize each of these scores to a mean of 50 and an SD of 10, with a range between 0 and 100 [15]. Most scores fall between scores of 20 and 80. In addition to these 5 PROMIS scores, a score related to exercise frequency and intensity was assessed through the in-app Godin Leisure-Time Physical Activity Questionnaire [28,29]. For the Godin score, a patient scoring ≥24 was considered active, and a patient scoring <24 was considered inactive as a generalization [30]. PROMIS computer adaptive testings have previously shown efficacy in previous studies, in addition to low barriers to form completion [14].

“Symptom” (pain interference, fatigue, and sleep disturbance) scores of 60 (1 SD above the average of 50) or higher were defined as moderate to severe symptom severity [31]. Similarly, “function” (physical function and social activity) scores of 40 (1 SD below the average of 50) or below were defined as moderate to severe symptom severity, meaning less functional ability than normal. In this study, multiclass classification techniques were used to classify PRO state or score transitions over time, and binary classification techniques were used to determine whether patients’ PRO scores were above or below the critical threshold for at least moderate symptom or functional severity.

### Data Imputation and Preprocessing

Missing data are a concern in any study that involves consistent patient adherence to a study protocol over time. In this study, missing activity tracker data could have resulted from patients either forgetting to wear their devices or removing them for charging, and missing weekly PRO scores could have resulted from patients forgetting to fill out their PROMIS surveys on a given week. Patients may have also gotten tired of completing PRO surveys consistently as the study progressed.

At first, prior to any data preprocessing, each of the 15 smartwatch-derived feature columns consisted of daily aggregated measurements. In other words, the original data set had 12 weeks (84 days) of data for each of the 278 originally qualifying patients in total, with each row corresponding to a given day’s measurements of the activity tracker features. However, weekly PRO measurements only presented themselves once a week, and this meant that we needed to aggregate data weekly rather than daily. To address this, we preprocessed the data set such that each of the 15 physical feature columns was split into 7 columns corresponding to the 7 days in a week. For instance, the DailyTotalSleepMins column, which measured how many minutes the patient was asleep in a given day, was split into 7 “unmelted” columns (eg, DailyTotalSleepMins1, DailyTotalSleepMins2, …, DailyTotalSleepMins7) such that there would not be any missing PRO values for that given week if the patient filled out all of their PROMIS surveys. It was first decided that the criteria for dropping a given week of data prior to model training and evaluation would be if there were at least 3 days of missing data for any of the 15 Fitbit-derived features. We excluded patients from our data set who had more than 2 weeks of dropped rows at any time during the 12-week study period. Based on these specifications, 59 of the 278 originally qualifying patients were excluded, and thus, 219 patients ended up remaining in the data set. In addition, prior to training our machine learning models to generate predictions for each of the PRO scores, we imputed remaining missing feature data based on the corresponding feature means from the previous week.

To establish distinct states that correspond to PRO score ranges, 8 “bins” of scores were created within a range of 5 (0.5 SD) units around the score’s established threshold for a given PRO score. Specifically, we created 3 score groupings below the threshold and 3 score groupings above the threshold, with 2 “catch-all” bins that grouped together all scores below and above the 6 central score groupings. As an example, this grouping process resulted in 8 unique states for the sleep disturbance PRO score: 40 (grouped all scores that were below 40 together), 45, 50, 55, 60 (the threshold grouping), 65, 70, and 75 (grouped all scores that were above 75 together). We manually merged bins that had less than 40 observations with the adjacent bins to avoid sparsity. When it came to measuring the success of the machine learning models themselves, we trained and evaluated each model 10 times and outputted the average metrics of those 10 rounds since the random seed of each iteration was different.

### Independent Per-Week RF Model

**Figure 1:**
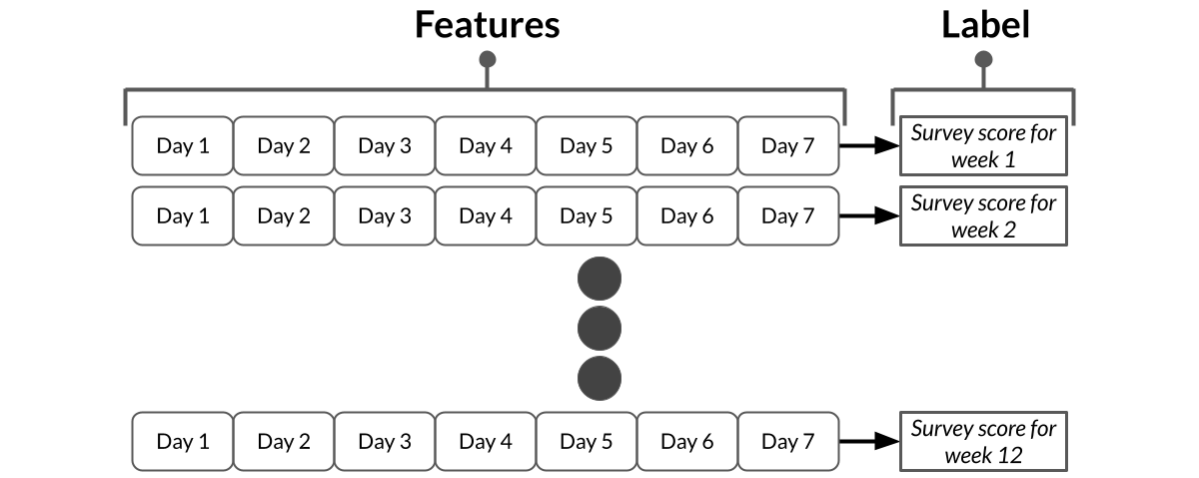
Random forest schematics.

A naïve approach to model creation would involve treating each week independently, as PROMIS survey scores were generated on a weekly basis. A 1-subject example of the independent model is illustrated in Figure 1, as there would be 12 weeks of evaluable data that are treated as independent observations. All the features for the 7 days of the week were appended into a single feature vector, which was then used to train the machine learning models for the task of PRO score classification. Ensemble methods such as RF, Adaboost, and gradient-boosted regression trees have previously been shown to be robust over unbalanced data sets and are capable of generating better classification accuracies compared to other independent machine learning models [32]. In this particular set of analyses, the RF model was used to establish a baseline performance that the HMM could potentially improve on. Through hyperparameter tuning, we determined that each RF instance should consist of 100 estimators (decision trees), use the Gini Index as criteria for splitting, allow a maximum depth of 25 to prevent overfitting, and require at least 10 training samples as the minimum threshold for splitting. In addition, through use of the RF model, we were able to assess the relative importance of the 15 different activity tracker–derived features in carrying out PRO score classification.

### HMM With a Forward Algorithm

**Figure 2:**
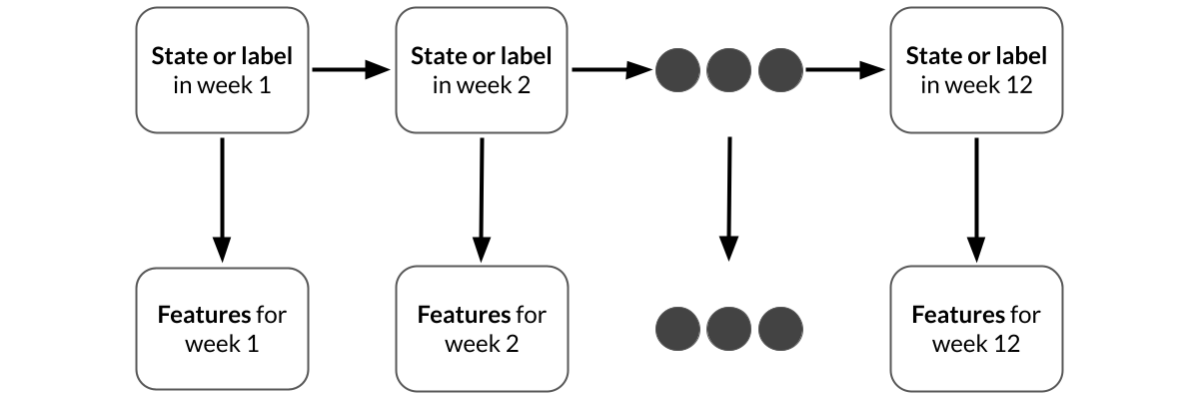
Hidden Markov model schematics.

A significant shortcoming of models that treat each week independently (such as the RF) is that they do not take temporal factors into account, or how previous PRO score states influence the current PRO score state. The use of the HMM addresses this weakness and effectively incorporates temporal correlations of PRO score states across weeks. As illustrated in Figure 2, the HMM’s state at each week corresponded to the PRO score state for that given week, with the activity tracker–derived features treated as observations. Following the score grouping detailed in the Data Imputation and Preprocessing section, we indexed each of the unique scores or states starting from 1 and created transition matrices by counting the *S* state transitions from week to week. These transition matrices were then normalized based on the Gaussian standard distribution and were used to generate label predictions in the testing and evaluation phase from the prediction probabilities generated by the RF model.

The forward algorithm computed the probabilities across states at time *t*, with the maximum computed probability representing the state classification,



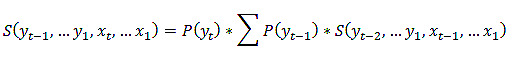



where the weekly PRO score was treated as state *y_t_* with observations of features *x_t_* [33]. The emission probability, *P*(*x_t_*|*y_t_*), computed the probability of the observed feature vector *x_t_* given state *y_t_*, computed from the RF classifier and *P*(*y_t_*).



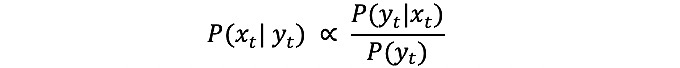



At the first step, the transition probability distribution is undefined, so the state probability was defined as:

*S*(*y*_1_|*x*_1_)∝*P*(*x*_1_|*y*_1_)*P*(*y*_1_)

For the task of binary classification, states were binarized according to whether or not they were normal or mild (ie, within 10 units of the US population mean of 50) or moderate or severe, based on the thresholds described in the PRO Measures section. Because dichotomizing PRO score values loses some information and adds noise, multiclass classification of PRO score states was conducted as well without the binarization process. For the binary classification task (distinguishing between a “normal” and “severe” PRO score), we chose to use the receiving operating characteristic area under the curve (ROC-AUC) metric, as it would give us a comprehensive understanding of the relationship between the true and false positive rates as we vary the classification threshold. For the multiclass classification task (PRO state prediction), we chose to use the Pearson correlation coefficient metric to evaluate the statistical relationship between the models’ predicted state labels and the true state labels, as well as the quadratic weighted Cohen κ coefficient to measure agreement or consistency between predicted state labels and true state labels. For each PRO score, the RF and HMM were trained and evaluated 10 times each to establish robustness in the observed metrics.

### Role of the Funding Source

The funders of the study had no role in the data analysis, data interpretation, or writing of this report.

### Ethics Approval

For all patient-level data used in this study, we obtained patients’ written informed consent. Patients’ consent statements have been kept on file. Our research activities related to this study were governed by the University of Alabama at Birmingham IRB (160708003).

## Results

Between the HMM and RF models, the Pearson correlation coefficient results from multiclass classification of PRO scores are summarized in Table 2, quadratic-weighted Cohen κ coefficient results from multiclass classification of PRO scores are summarized in Table 3, and ROC-AUC results from binary classification of PRO scores are summarized in Table 4.

For the multiclass classification task, we observed a consistent trend of the HMMs attaining significantly higher Pearson correlation coefficients and quadratic-weighted Cohen κ coefficients than the corresponding RF across all PRO scores. Using the HMM, the highest Pearson correlation coefficient and quadratic-weighted Cohen κ coefficient values were 0.479 and 0.471, respectively, for classifying the weekly physical function PRO score, and the lowest values were 0.204 and 0.200, respectively, for classifying the weekly sleep disturbance PRO score.

Regarding the binary classification task of distinguishing normal or mild PRO scores from moderate or severe PRO scores, the HMM outperformed the RF model to a significant (*P*<.05) degree across all PRO scores. The highest overall ROC-AUC score was achieved for the weekly exercise PRO score with the HMM and RF models attaining ROC-AUC scores of 0.750 and 0.742, respectively. On the other hand, the lowest overall ROC-AUC scores were 0.653 and 0.637, respectively, for the weekly sleep disturbance PRO score. Relative feature importance for the physical function and exercise PROs was also recorded through the evaluation process, and relative feature importance levels are depicted in Table 5. The top 3 important features for the physical function PRO, in order of relative importance, were total steps, total activity minutes, and minimum heart rate, and the top 3 important features for the exercise PRO were total steps, very (intense) activity minutes, and minimum heart rate. Intuitively, it makes sense that these features played an important role in classification for these PRO scores, as these features especially would correlate strongly with an individual’s overall physical activity and exercise levels over time.

**Table 2:** Pearson correlation coefficient results for multiclass classification of PRO^a^ scores.

Label	HMM^b^, mean (SD)	RF^c^, mean (SD)
Weekly physical function PRO score	0.479^d^ (0.0184)	0.381 (0.0141)
Weekly exercise score	0.434^d^ (0.0112)	0.366 (0.0091)
Weekly fatigue PRO score	0.376^d^ (0.0309)	0.235 (0.0167)
Weekly pain interference PRO score	0.330^d^ (0.0237)	0.193 (0.0218)
Weekly social activity score	0.316^d^ (0.0331)	0.160 (0.0310)
Weekly sleep disturbance PRO score	0.204^d^ (0.0418)	0.165 (0.0173)

^a^PRO: patient-reported outcome.

^b^HMM: hidden Markov model.

^c^RF: random forest.

^d^Denotes significant improvement over RF.

**Table 3:** Quadratic-weighted Cohen κ results for multiclass classification of PRO^a^ scores.

Label	HMM^b^, mean (SD)	RF^c^, mean (SD)
Weekly physical function PRO score	0.471^d^ (0.0201)	0.352 (0.0134)
Weekly exercise score	0.393^d^ (0.0184)	0.352 (0.0131)
Weekly fatigue PRO score	0.370^d^ (0.0305)	0.181 (0.0161)
Weekly pain interference PRO score	0.322^d^ (0.0251)	0.161 (0.0193)
Weekly social activity score	0.313^d^ (0.0326)	0.111 (0.0216)
Weekly sleep disturbance PRO score	0.200^d^ (0.0407)	0.114 (0.0112)

^a^PRO: patient-reported outcome.

^b^HMM: hidden Markov model.

^c^RF: random forest.

^d^Denotes significant improvement over RF.

**Table 4:** Area under the receiving operating characteristic curve values of binary classification of PRO^a^ scores.

Label	HMM^b^, mean (SD)	RF^c^, mean (SD)
Weekly exercise score	0.750^d^ (0.0105)	0.742 (0.0091)
Weekly physical function PRO score	0.745^d^ (0.0119)	0.736 (0.0116)
Weekly fatigue PRO score	0.699^d^ (0.0138)	0.682 (0.0078)
Weekly social activity score	0.673^d^ (0.0221)	0.664 (0.0190)
Weekly pain interference PRO score	0.655^d^ (0.0082)	0.642 (0.0126)
Weekly sleep disturbance PRO score	0.653^d^ (0.0188)	0.637 (0.0138)

^a^PRO: patient-reported outcome.

^b^HMM: hidden Markov model.

^c^RF: random forest.

^d^Denotes significant improvement over RF.

**Table 5:** Feature importances for physical function and exercise PRO^a^ scores, ranked by relative importance for classification.

Physical function PRO	Exercise PRO
Total steps (0.0983)	Total steps (0.1065)
Total activity minutes (0.0764)	Very (intense) activity minutes (0.0925)
Minimum HR^b^ (0.0738)	Minimum HR (0.0751)
Maximum HR (0.0703)	Total activity minutes (0.0685)
Total calories (0.0672)	Total sleep minutes (0.0664)
Light activity minutes (0.0647)	Light sleep minutes (0.0608)
Total sleep minutes (0.0624)	Total calories (0.0601)
Light sleep minutes (0.0620)	REM^c^ sleep minutes (0.0598)
Deep sleep minutes (0.0608)	Deep sleep minutes (0.0586)
Sedentary activity (0.0588)	Light activity minutes (0.0567)
Wake minutes during sleep (0.0579)	Maximum HR (0.0556)
REM sleep minutes (0.0565)	Wake minutes during sleep (0.0552)
Very (intense) activity minutes (0.0391)	Sedentary activity minutes (0.0513)
Moderate activity minutes (0.0338)	Moderate activity minutes (0.0502)
Total (Fitbit) wearing minutes (0.0226)	Total (Fitbit) wearing minutes (0.0250)

^a^PRO: patient-reported outcome.

^b^HR: heart rate.

^c^REM**:** rapid eye movement.

## Discussion

### Principal Findings

In this analysis of passive data collected by a health activity tracker device (Fitbit Versa 2), we found generally high correlations with weekly PROMIS, other PROs related to RA, and self-reported exercise. Correlations between the passive data and the actively contributed PROs were highest for weekly physical function score, weekly fatigue score, and the exercise score from the Godin Leisure Time Exercise Instrument. This might be expected given that the health activity tracker provides the most information about actigraphy. Performance metrics for the multiclass and binary tasks of classifying PRO scores were significantly higher for the HMM compared to the RF. This general trend makes sense, as the HMM essentially builds from the independent RF model by factoring in sequential state changes when it comes to predicting a PRO class for a given week. A similar study, focused on classifying PRO scores in patients with stable ischemic heart disease, generally observed similar results, but in that study, the independent RF model outperformed the HMM for some of the PRO scores [23]. The results of our study could indicate that the HMM’s usage could vary from disease to disease and that it is effective in using activity tracker data to predict changes in RA symptom progression or improvement in particular. Although our results show promise for the use of temporal models for clinical applications such as passive remote monitoring, there is always room for future improvement. Even though the HMMs consistently outperformed the independent RF models for the multiclass classification task, the average Pearson correlation coefficient of HMM predictions and true labels across all PRO scores was 0.357, and the average quadratic weighted Cohen κ coefficient was 0.345. Although these values should not be regarded as “poor,” they suggest that physical activity trackers may not capture all the relevant information needed to make accurate predictions about changes in different aspects of a patient’s disease state. As we saw, our models perform relatively better for measures related to actigraphy since that type of data was directly captured by the activity tracker. Data about mental or emotional states were not incorporated into our models, which might be possible by examining heart rate variability or electrodermal activity and could potentially help with PRO score classification for emotional states. Another objective for future studies would be to further increase patient adherence rates and reduce the percentage of missing data, as this is generally one of the largest obstacles for accurate mHealth telemonitoring, including our study [9]. With more precise data about a patient’s state at a given point in time, statistical models could be better tuned to classify PRO score states over time and thus improve overall accuracy metrics and usefulness in a clinical setting. In the future, similar studies could also be carried out using deep learning methods such as long-short-term-memory networks (a class of recurrent neural networks), which have both shown prowess when dealing with sequential clinical data and missing data [34,35]. These kinds of approaches effectively model varying length sequences and capture long-range dependencies and could serve as another approach to classifying PRO scores over time [34]. A caveat is that these types of deep learning methods require a large amount of training data (ie, more patients in the clinical study) in order to perform robustly, which was not available for this study. As the efficacy of collecting clinical data from patients continues to improve over the next few years, such complex approaches will become more and more attainable and practical.

PROMIS instruments themselves could also be a source of potential improvement for model development. Even though recorded PROs have face validity and provide useful information about the patient experience, some doubts revolve around how some PRO scores may be partially redundant with each other and may not serve as the most effective way to inform treatment decisions [36]. In addition, patients may experience response fatigue when taking PROMIS surveys due to their question-by-question format, which could lead to measurement error and misclassification [37,38]. Improving the patient experience while completing a more limited set of PROMIS surveys could be a way to address the issues of low completion rates and reduce response fatigue that may take place in clinical studies such as this one. In a clinical setting, we envision a hybrid approach where PRO data collection is complemented by passive actigraphy measures, with sophisticated algorithms applied to both, such as those that we used. As advances in technology such as more widespread availability of the internet and mobile devices become more prevalent, patient accessibility increases, and thus, patients’ ability to participate in studies related to PRO score classification and related clinical intervention applications can improve [4].

A related use case for these kinds of models would be to detect worsening from a patient’s baseline state over time. In a clinical setting, it would serve greater usage to notify physicians when a patient’s state has significantly worsened from an initially measured state (ie, after the start of medication administration or a previous physician checkup). In our analysis, patients’ initial state (time 0) was a random, unimportant time. However, if “time 0” was instead marking the start of a new drug treatment, then there would be an underlying slope of PRO score trends and an expected trajectory of score improvements. In a clinical study such as this, our models would be trained and evaluated to detect the first week of significant decrease in PRO scores from baseline or initial scores over the study period.

We acknowledge several study features that are useful to contextualize this work. First, based on the demographics of the ArthritisPower registry, participants are predominantly White and have higher amounts of education (ie, at least some college), so these results may or may not generalize to other demographic groups, and they may be influenced by other social determinants of health. Ongoing work in the registry is explicitly recruiting a more diverse community of patients with respect to these factors. Our sample size of only 219 patients was admittedly modest to train a machine learning model, but the number of patients recruited was constrained by project resources. With larger sample sizes or the ability to pool across several similar data sources (ie, from other ongoing studies being conducted in the ArthritisPower registry), additional features may emerge as important. As described earlier, we also recognize that deep learning models may provide performance improvements in some settings, which also would be facilitated by a larger sample size.

### Conclusions

Machine learning methods can be used to classify self-reported PRO scores over time in patients with RA, and temporal methods such as HMM have been shown to consistently outperform independent models such as the RF for binary and multiclass classification tasks. Our study indicates that passively generated data from activity trackers can be useful in a machine learning environment to classify health status over time and that additional research should be conducted to further validate the use of such frameworks for clinical applications such as remote health monitoring and early intervention for patients with chronic illnesses. Encouragingly, we note that clinical use of monitored mobile apps and passive biosensor devices are now reimbursable by insurance in real-world settings. Since 2019, Medicare and other commercial insurance companies now compensate providers for care provided through remote physiologic monitoring and remote therapeutic monitoring programs [39]. These potentially transformative opportunities offer the potential to accelerate the adoption of approaches like ours and move remote monitoring out of research settings and into routine, clinical settings where they can positively impact care.

## Abbreviations

DIGITAL: Digital Tracking of Arthritis Longitudinally
HMM: hidden Markov model
mHealth: mobile health
PRO: patient-reported outcome
PROMIS: Patient-Reported Outcomes Measurement Information System
RA: rheumatoid arthritis
RF: random forest
ROC-AUC: receiving operating characteristic area under the curve
UCLA: University of California, Los Angeles

## Data Availability

Data collected for this study can be made available upon request.
